# Masking, social context and perceived stress in autistic adults: An ecological momentary assessment study

**DOI:** 10.1177/13623613251353358

**Published:** 2025-07-09

**Authors:** Anke M Scheeren, Smiddy Nieuwenhuis, Laura Crane, Yvette Roke, Sander Begeer

**Affiliations:** 1 Vrije Universiteit Amsterdam, The Netherlands; 2 Amsterdam Public Health Research Institute, The Netherlands; 3 University of Birmingham, UK; 4 GGz Centraal, The Netherlands

**Keywords:** adults, autism, camouflaging, masking, stress

## Abstract

Masking may entail the suppression of autistic traits by autistic individuals. Thus far, research indicates a negative impact of autistic masking on mental health, but this is largely based on retrospective surveys. In this study, we used ecological momentary assessment to examine real-time associations between social context (i.e. presence of (non-)autistic others), masking, and perceived stress in everyday life among a sample of autistic adults. Ecological momentary assessment data were collected via a smartphone application for 87 autistic individuals (58 females; *M* age = 48; age range: 17–68). In line with the hypotheses, repeated measures analyses of variance and linear mixed models indicated that (1) participants reported masking significantly less when they were alone compared with when others were present, (2) participants masked significantly more when non-autistic others were present compared with autistic others, and (3) more masking was associated with a concurrent higher level of perceived stress. Autistic adults reported they could be more themselves among autistic peers and reduced masking was associated with reduced stress. These ecological momentary assessment study findings provide ecological validity to the potential stressful impact of masking in the daily lives of autistic adults.

**Lay abstract:**

Autistic people may try to hide their autistic traits in order to fit in. This is called autistic masking. Survey research suggests that autistic masking may have a negative effect on the mental well-being of autistic people. Yet, survey research has limitations, because people may not remember or may not accurately report how much they masked and how they felt in the past. Therefore, in this study, we asked autistic adults to use a smartphone app to report with whom they were (with or without autistic people), if they could be themselves (degree of masking), and how stressed they felt during the past 4 h. Participants reported this information multiple times over a period of 28 days. In total, 87 autistic adults participated (58 females; age range: 17–68). In line with our expectations, (1) participants masked less when they were alone compared with when others were present, (2) participants masked more when non-autistic others were present compared with autistic others, and (3) more masking was linked with the experience of more stress in the same moment. Autistic adults reported they could be more themselves among other autistic individuals. Also, less masking was associated with less stress. Our study shows the everyday reality of stress during masking experienced by autistic adults.

*Be yourself—there are already so many others.*

Loesje

Autistic individuals^1^ have described using specific behavioral and cognitive strategies to fit in a predominantly non-autistic society (Cook et al., 2021). In the literature, this phenomenon is often called “camouflaging,” but it may also be termed “masking” or “compensation.” Following autistic community preferences, and as suggested by Pearson and Rose (2021), we use the term “masking” here. Adapting behavior to fit in is not unique to autistic people (Jorgenson et al., 2020; van der Putten, Mol, Groenman, et al., 2024). However, while most, if not all, people try to shape the impression others have of them, the motives and costs of impression management may differ from autistic masking (Ai et al., 2022; Pearson & Rose, 2021; Scheeren et al., 2016). Whereas impression management typically consists of highlighting a specific, advantageous side of oneself in order to gain something, masking may (also) entail hiding an aspect of oneself to prevent a poor outcome such as stigmatization (Ai et al., 2022; Hull et al., 2017; Pearson & Rose, 2021; Perry et al., 2022). Moreover, masking may come at the expense of poor mental health (Cook et al., 2021). In this study, we focus on real-time associations between social context, masking, and perceived stress in autistic adults in daily life situations.

Given that autistic masking exists by virtue of an audience, and more specifically, a non-autistic audience, it is likely that autistic individuals mask most in the company of non-autistic others and mask least when alone. Indeed, qualitative reports suggest that individuals with autism are more likely to mask when they feel like they cannot be their authentic self in the presence of others (Cook et al., 2024; Watts et al., 2024), and they may drop the mask as soon as they are alone in the safety of their own home (Lewis & Stevens, 2023). There are various possible reasons why autistic people may mask more in the company of specifically non-autistic people. First, autistic individuals may experience minority stress (Botha & Frost, 2020) and fear of being “othered” and stigmatized (Ai et al., 2022; Hull et al., 2017; Pearson & Rose, 2021; Perry et al., 2022). Second, in line with the double-empathy problem (Milton, 2012), it may be easier for autistic adults to empathize, socialize, and communicate with each other rather than with non-autistic peers, because of similarities in information processing and communication style. Indeed, in empirical studies, adults with autism show more effective communication skills (Crompton et al., 2020), more confidence in their social skills (Camus et al., 2024), and more self-disclosure (Morrison et al., 2020) during social interactions with autistic than with non-autistic individuals. Increased empathy and increased social self-efficacy among autistic people may result in less masking and thus a more complete and authentic display of oneself in the company of other autistic people (Cook et al., 2024; Watts et al., 2024). Sociocultural norms may also impact the degree and function of masking during social interactions. For example, Funawatari et al. (2024) recently found that Japanese autistic adults masked more in hypothetical situations with other people with autistic traits than with non-autistic others. This counterintuitive finding was attributed to autistic adults seeking social connections with like-minded peers, thus masking in the presence of those with autistic traits in order to convey the “best” version of themselves.

Compared with non-autistic adults, autistic adults generally report experiencing more mental health problems and higher levels of perceived stress (Bishop-Fitzpatrick et al., 2017; Hirvikoski & Blomqvist, 2015; Lai et al., 2019; van der Linden et al., 2021). Masking could contribute to these mental health problems and psychological stress (Bradley et al., 2021). In a systematic review by Cook et al. (2021), eight out of 10 included studies found significant associations between masking and reduced mental health in autistic adults, usually indicated by higher levels of anxiety, depression, and/or psychological stress on retrospective questionnaires. Cage and Troxell-Whitman (2019) compared self-reported masking in autistic adults in England across hypothetical formal (e.g. colleagues) and informal (e.g. family and friends) social contexts, with highest stress levels reported by those masking strongly in either one or both formal and informal social contexts. Qualitative studies also emphasize the psychological stress that comes with masking (Field et al., 2024; Hull et al., 2017). Many autistic individuals express frantically monitoring social situations, worrying about whether they are doing it “right” and the potential backlash of social errors (Field et al., 2024; Hull et al., 2017). More recent quantitative research, not included in the systematic review of Cook et al. (2021), suggests that the link between masking and mental health may in fact be relatively modest for a majority of adults with autism, but strong for a subgroup with heightened autism traits or negative affect (van der Putten, Mol, Radhoe, et al., 2024).

The impact of masking on stress may also differ for different sexes/genders. Most studies suggest that autistic women generally engage in more masking and potentially more convincing masking than autistic men, as proxies attribute fewer autistic traits to autistic women than women attribute to themselves (Cook et al., 2021). Stronger societal pressure for women to “be social” may lead women to mask their autistic tendencies or behaviors (Pearson & Rose, 2021). More (convincing) masking by women likely results in delayed recognition and diagnosis of autism (Milner et al., 2024), an underestimation of experienced difficulties (Field et al., 2024) and a lack of environmental adaptations to their needs, perpetuating challenging environments where autistic women feel the need to mask.

A methodological weakness of most quantitative masking studies has been their reliance on retrospective questionnaires and cross-sectional designs. Retrospective reports can be biased, as people may have difficulties remembering or estimating how stressed or anxious they felt in the past (e.g. past month). A potential solution to this problem is to let people evaluate their stress level in the moment, as is the case in ecological momentary assessment (EMA) studies. EMA studies enable temporary contextual and personal factors in everyday life to be measured relatively easily with smartphone applications. A further benefit of EMA studies compared with retrospective, cross-sectional studies is that they enable the study of temporal associations: a factor (e.g. masking) at one point in time may have an effect (e.g. perceived stress) at a subsequent moment. In the current EMA study, we therefore use a smartphone application to repeatedly assess participants’ perceived stress in the moment. This research design allows for the examination of real-time and temporal associations between social context, masking, and perceived stress in everyday life.

To date, relatively few EMA studies have addressed the social experiences of autistic individuals. The limited work that does exist has shown similarities between autistic and non-autistic adolescents and young adults in terms of the number of social interactions they have and the amount of time they spend alone (Feller et al., 2023; Gerber et al., 2019; Hintzen et al., 2010), although autistic individuals seem to spend more time with their family or people they live with and less time with their friends (Feller et al., 2023; Hintzen et al., 2010). In addition, studies have shown that autistic individuals generally felt more nervous and judged in the company of others compared with their non-autistic peers (Feller et al., 2023), which aligns with a fear of autism-related stigmatization found in other studies (Bradley et al., 2021; Hull et al., 2017). Relatedly, Dallman et al. (2022) were able to predict the positive and negative affect of autistic male adolescents based on their concurrent enjoyment of a social interaction. To our knowledge, there have not been any EMA studies examining the social context and impact of masking on the mental health of individuals with autism.

In the current EMA study, we examined real-time associations between masking, social context, and perceived stress in the everyday lives of autistic adults. Following the literature, we hypothesized that (1) autistic individuals engage in less masking when they are alone compared with when others are present, (2) autistic individuals engage in more masking when they are in the presence of non-autistic individuals as opposed to autistic individuals, and (3) a high degree of masking in the past 4 h would be associated with a high level of stress experienced during that same time period. In addition, we explored whether these associations were moderated by sex / gender and degree of autism traits. Finally, we conducted an exploratory analysis of the potential impact of masking at one timepoint on experienced stress at a following timepoint that same day.

## Methods

### Participants

Autistic adults were recruited via the Netherlands Autism Register (NAR), a research center following a large cohort of children and adults with autism in the Netherlands. Participants had a confirmed clinical autism spectrum disorder diagnosis that was established by a team of authorized professionals working independently from the NAR. All participants in this study were taking part in a broader randomized controlled trial (Spaargaren et al., 2025) examining the effectiveness of a stress monitoring app specifically developed for and in co-creation with autistic adults (Stress Autism Mate, SAM). This study was approved by the Scientific and Ethical Review Board of the Faculty of Behavioural and Movement Sciences of the Vrije Universiteit Amsterdam (VCWE-2023-024R1). Study set-up, hypotheses, and main analyses of the current study were preregistered at the Open Science Framework (https://osf.io/98jup). Data were included in the analyses when a participant completed at least 25% of all EMA assessments, which is in line with prior EMA studies (Feller et al., 2023; van der Linden et al., 2021). We followed the suggestion of Oleson et al. (2022) and favored the number of participants over the number of assessments per participant, to increase the statistical power of the analyses. This 25% criterion resulted in a final sample of 87 autistic participants ranging from 17.9 to 68.9 years (*M* = 48.46, *SD* = 12.78). Most (87%) were diagnosed with autism in adulthood and many (55%) obtained a higher education degree (see Table 1).

**Table 1.** Background characteristics of the sample.

	*N*	*M*	*SD*	Min	Max
Age	87	48.46	12.78	17.90	68.90
Mean age autism diagnosis	83	37.95	13.62	5.00	62.67
AQ-28 total (Autism Quotient)	87	84.57	9.93	63.00	109.00
	*n*	*%*			
Sex assigned at birth					
Male	29	33.3			
Female	58	66.9			
Self-identified gender					
Man	29	33.3			
Woman	47	54.0			
Non-binary / other	11	12.6			
Co-occurring conditions					
No	38	43.7			
Yes^a^	44	50.6			
Missing	5	5.8			
Educational degree					
High	48	55.2			
Middle	31	35.6			
Low	6	6.9			
Missing	2	2.3			

a Most commonly reported co-occurring conditions were depression (23.3%), attention deficit hyperactivity disorder (14.0%), and trauma/post-traumatic stress disorder (12.8%).

### Procedure

Following informed consent of the participants, their momentary assessment data were collected via SAM. SAM is a freely available smartphone app that regularly (2 to 4 times each day, depending on user preference) prompts users to fill in a brief survey concerning their activities and perceived stress in the past 4 h (for more information about SAM, please see Hoeberichts et al., 2024). After each survey, users receive immediate feedback on their overall level of perceived stress in the past 4 h and, if needed, they receive an automated suggestion of what they could do to reduce their stress. For the purpose of this study, the SAM survey was supplemented by questions about social context and masking. Users did not receive feedback on their degree of masking.

Participants were asked to use SAM for a period of 28 days. However, the app allows users to skip assessments on particular days (e.g. weekends). The user selects a preferred time for their first daily assessment, automatically followed by next assessment(s) in 4-h intervals that same day. If a user does not respond to the prompt to fill in the survey, they receive an automated second prompt after 30 min. The user then has another 30 min to fill in the survey.

### Measures

#### EMA data

*Social context* was assessed with two items containing four response options: (1) “In the past four hours, I was mainly . . . alone / with partner / with family or friends / with other people,” and (2) “In the past four hours, I was mainly with . . . people with autism / people without autism / a mixture of people with and without autism / not applicable, because I was alone.” For the current research aims, we combined these items and created the following categories: being alone / being with people with autism / being with people without autism or a mixture of people with and without autism. Only 35 participants reported that they had been in the sole company of other people with autism. Therefore, the sample to test Hypothesis 2 is smaller (*n* = 35).

*Masking* was assessed with one item: “In the past four hours, I could completely be myself.” Prior research indicates that autistic individuals mask less when they feel they can be their authentic selves (Cook et al., 2024). Response options were Yes, strongly (0), Yes, a little (1), No (2). “Yes, strongly” indicates little to no masking, whereas “No” indicates a high degree of masking. To test the main hypotheses, a masking percentage across all assessments was calculated: (masking sum score / total assessments × 2) × 100. For instance, a participant who filled in the SAM survey 14 times and who selected “Yes, a little” each time will have a masking sum score of 14 and a masking percentage of 50% ((14 / 28) × 100). A higher masking percentage indicates more masking.

*Perceived stress* was assessed with seven items regarding the presence and intensity of stress-related feelings and thoughts in the past 4 h (e.g. “Did you feel irritable?”). The content, phrasing, and possible answers to these questions of the SAM app were developed and validated in co-creation with autistic adults (Hoeberichts et al., 2024). Each stress item was measured on a 4-point Likert-type scale (0 = *no*, 1 = *yes but not more than normal*, 2 = *yes, more than normal*, and 3 = *yes, much more than normal*), creating a sum score of perceived stress ranging from 0 to 21. A higher score indicates a higher (than normal) level of perceived stress. In the current study sample, the internal consistency based on the individual centered items scores was high (α = 0.85).

#### Demographic information

*Co-occurring conditions* were measured with one multiple-choice item in the yearly online questionnaire of the NAR. Participants were asked to select any co-occurring psychiatric diagnosis from a list of diagnoses, including anxiety disorder, depression, and attention deficit hyperactivity disorder (ADHD). If a participant selected one or more diagnoses, this was coded 1. If a participant selected no additional diagnoses, this was coded 0.

*Sex* was assessed with one item asking participants about which sex was assigned at birth (male/female/sex could not be determined) and reported in their birth certificate.

*Gender* was assessed with one item where participants selected one option to describe the gender they identify with: man / woman / partly man, partly woman / neither man, nor woman / sometimes man, sometimes woman / don’t know (yet) / other / rather not say. These categories were recoded into three broad gender categories: man, woman, and non-binary/other genders.

#### Community involvement

The NAR regularly collaborates with autistic individuals and their families to shape future autism research. The NAR also has autistic team members involved in all stages of research. In this study, the SAM app, including items about past activities and stress, was developed in co-creation with autistic individuals to improve their stress management. Items regarding masking and social context were added to the SAM app for the purpose of this study, but these were not developed nor screened by an autistic panel.

#### Statistical analyses

All analyses were performed using IBM SPSS Statistics (Version 29) and Rstudio (Version 2023.06). The number of completed SAM assessments was highly variable between participants. Based on two SAM assessments per day across a period of 28 days, participants’ EMA data were deemed complete (100%) when these contained 56 (2 × 28) assessments or more. Following Oleson et al. (2022), we favored the number of participants over the number of assessments per participant, and therefore included participants with a minimum of 25% completed assessments (0.25 × 56 assessments = 14 assessments).

We used chi-square analyses and independent *t* tests to examine whether participants included in the main analyses (with a minimum of 14 assessments) differed from excluded participants (with fewer than 14 assessments) by comparing their age, gender ratio, degree of autism traits, and presence of co-occurring conditions. This same procedure was followed to compare the group that had been in the sole company of other autistic people at least once (to test Hypothesis 2) and the group who had not.

#### Main analyses

We performed analyses in SPSS on aggregated data to capture stable, broad patterns of masking and stress across timepoints. To test the first two hypotheses, we compared the aggregated percentages of masking between social contexts within participants using a repeated measures analysis of variance (RM ANOVA). Simple contrasts were used to investigate whether the degree of masking when alone differs from masking in the company of others (first hypothesis), and repeated contrasts were used to test whether the degree of masking in the company of autistic others differs from degree of masking in a neurotypical or mixed neurotype context (second hypothesis). We used a hierarchical multiple linear regression analysis to predict level of perceived stress based on the aggregated percentage of masking (third hypothesis). Our model-building strategy followed a theoretically driven sequence. In the first step, we added the covariate co-occurring mental health conditions. Presence of co-occurring conditions was added as a control variable in all analyses because prior research indicates that many autistic individuals have co-occurring conditions such as ADHD (Hossain et al., 2020; Lai et al., 2019) and these conditions have also been associated with masking (van der Putten, Mol, Groenman, et al., 2024) and lower well-being (Sonuga-Barke et al., 2023). In the next step, we added main effects while controlling for the covariate. Finally, we incorporated relevant interaction terms. This approach allowed us to systematically evaluate how each set of predictors contributed to explained variance in masking and stress. In all cases, we interpreted the parameters of the final model.

Effect sizes of the RM ANOVA are partial eta squared $(\eta_{p}^{2}),$ with $\eta_{p}^{2}$ ⩾ 0.01 considered small, $\eta_{p}^{2}$ ⩾ 0.06 medium, and $\eta_{p}^{2}$ ⩾ 0.14 large (Richardson, 2011). Amount of explained variance (*R*^2^) in the linear regression analysis is used as an effect size indicator, with a small (*R*^2^ ⩾ 0.02), medium (*R*^2^ ⩾ 0.13), and large effect (*R*^2^ ⩾ 0.26; Cohen, 1988). The significance level is set at *p* < 0.05 (two-tailed) for all analyses.

#### Exploratory analyses

Taking into account the nested structure of the data (repeated assessments per participant), we used Linear Mixed Modeling (LMM) to (1) test the robustness of our findings as shown in the main analyses, (2) examine the moderating role of sex assigned at birth (male/female) or self-identified gender (man/woman), (3) assess how masking is related to stress 4 h later that same day, and (4) test the moderating role of autism traits in the association between masking and stress. In all analyses, we controlled for the presence of co-occurring conditions. When predicting stress levels based on prior masking, we controlled for the current degree of masking, as we were specifically interested in the association with prior masking.

For masking, we added both the variable of masking that reflects the within-subject variability of masking (masking centered around the individual mean) and the between-subject variability of masking (mean masking). With this, we were able to investigate both the effects of day-to-day fluctuations in masking as well as the broader patterns of masking across participants.

We performed LMM with the nlme package in R when the residuals of the outcome variable were normally distributed. In all nlme analyses, we used restricted maximum likelihood ratios and the first-order autoregressive (AR1) covariance structure to account for the repeated measures. When the residuals were non-normally distributed, we used a generalized LMM (GLMM) with a Poisson or negative binomial distribution to handle the skewness. We included a random intercept at the participant level in all analyses. Significance level was set at *p* < 0.05 (two-tailed).

## Results

### Selective attrition check

Participants with a minimum of 25% assessment rate were included in the main analyses (*n* = 87) and did not significantly differ from excluded participants (*n* = 43) in terms of gender or sex (self-identified gender: χ^2^(2) = 0.16, *p* = 0.925; sex assigned at birth: χ^2^(1) = 0.009, *p* = 0.926), presence of co-occurring conditions (χ^2^(2) = 0.14, *p* = 0.709), age *t*(*df*) = 0.32(125), *p* = 0.749), and degree of autism traits as measured with the Autism-Spectrum Quotient–28 (*t*(*df*) = –0.34(124), *p* = 0.738). These results suggest that the inclusion criteria for the analyses did not introduce substantial bias with respect to these key demographic and clinical characteristics.

Furthermore, the sample included in the analysis to assess Hypothesis 2 (participants who reported that they had been in the sole presence of other autistics) did not differ on these characteristics from those that were excluded from the analysis (self-identified gender: χ^2^(2) = 2.74, *p* = 0.254; sex assigned at birth: χ^2^(1) = 0.81, *p* = 0.369; presence of co-occurring conditions: χ^2^(2) = 0.77, *p* = 0.380; age: *t*(*df*) = –0.99(85), *p* = 0.326; degree of autism traits: *t*(*df*) = 1.52(84), *p* = 0.132).

### Main analyses

Regarding Hypothesis 1, an RM ANOVA with social context (being alone vs. with others) as independent variable, degree of masking as dependent variable, and presence of co-occurring conditions as control variable showed a significant and large-effect difference between masking in the alone context (*M* = 16.05%, *SD* = 17.64%) and in the context with others (*M* = 36.07%, *SD* = 22.09%, *F*(1, 86) = 83.16, *p* < 0.001, $\eta_{p}^{2}$ = 0.51). As hypothesized, autistic adults masked less when alone.

In line with the second hypothesis, an RM ANOVA revealed that participants masked significantly more in the company of non-autistics or a mixed neurotype group (*M* = 39.60%, *SD* = 23.54%) compared with when they are in the company of other autistic adults (*M* = 20.39%, *SD* = 24.86%, *F*(1, 31) = 15.90, *p* < 0.001, $\eta_{p}^{2}$ = 0.34). In addition, although not part of our hypothesis, degree of masking was similar across the non-autistic (*M* = 36.8%) and mixed neurotype context (*M* = 36.1%).

As predicted in the third hypothesis, there was a significant positive relation between percentage of masking and perceived stress scores after controlling for the presence of co-occurring conditions (*b* = 0.08. *SE* = 0.01, β = 0.56, *t* = 6.05, *p* < 0.001). This indicates that more masking is associated with higher perceived stress scores. Notably, 32% of the variance in stress scores could be explained by masking alone, indicating a large effect. Important to note is that the values of stress were highly skewed, which is a common phenomenon with stress measures in EMA analyses.

#### Power analyses

Post hoc power analyses were conducted based on effect sizes derived from SPSS output. For Hypotheses 1 and 2, partial eta squared $\left(\eta_{p}^{2}\right)$ was converted to Cohen’s *f* using the standard transformation. For Hypothesis 3, the effect size was calculated using Cohen’s *f*² based on the change in *R*^2^ from the regression model. All three analyses indicated very high power (⩾0.95), with particularly high estimates for Hypotheses 1 and 3 (power ≈ 1.00). While these values suggest sufficient power, the effect sizes may be overestimated, especially given the small sample size in Hypothesis 2 and the known sensitivity of partial η² to sample fluctuations.

### Exploratory analyses

#### Calculation of intraclass correlation coefficient

The intraclass correlation coefficient (ICC) was calculated to quantify the proportion of variance attributable to between-person differences versus within-person fluctuations, thereby confirming the appropriateness of multilevel modeling and providing insight into the stability of masking behaviors across assessments. The ICC of masking is 0.28, indicating that 28% of total variance in the masking variable is attributable to between-subject differences, while the remaining 72% of the variance reflects within-subject variability. The ICC of perceived stress was 0.11, indicating that 11% of the variance is between-subject variance, while 89% is within-subject. In addition, the ICC for subsequent stress scores is 0.07, meaning that 7% of the variance in subsequent stress is between subjects and 93% within subjects. These relatively low ICCs indicate that any significant associations found in the LMMs can be largely attributed to how individuals vary across different contexts and time points, rather than stable differences between individuals.

#### Associations with age

Given the broad age range of our adult sample, we checked whether age was related to masking or stress across the full sample. These exploratory analyses showed no significant associations between age and masking (*p* = 0.33) or age and perceived stress (*p* = 0.12). Based on this, and to avoid unnecessarily complicating our statistical models, age was not included as a covariate in the LMMs.

#### Main hypotheses using LMM

With regard to the first and second hypotheses, LMM findings were similar to those of the prior RM ANOVAs, that is, autistic adults reported masking more when with others (vs. when alone; *b* = 0.40, *SE* = 0.02, *t*(*df*) = 24.50(4703), *p* < 0.001) and reported masking less when with autistic others (vs. with non-autistic others or a mixed neurotype group; *b* = –0.11, *SE* = 0.07, *t*(*df*) = –2.37(4702), *p* = 0.017).

To test the third hypothesis, we used a negative binomial generalized linear mixed model (nbGLMM) because stress scores were positively skewed. Similar to the prior findings based on the aggregated data, this analysis confirmed a significant positive association between masking and stress at the same time. This was found for both the within-subject variable of masking (*b* = 0.40, *SE* = 0.02, *z* = 20.33, *p* < 0.001) and the between-subject variable of masking (*b* = 1.40, *SE* = 0.22, *z* = 6.24, *p* < 0.001) on stress at the same time. These findings suggest that both day-to-day variation in masking within participants (within-subjects effect) and differences in average levels of masking between participants (between-subjects effect) were significantly associated with stress at the same time.

#### First hypothesis: sex/gender moderation

**Figure 1.**
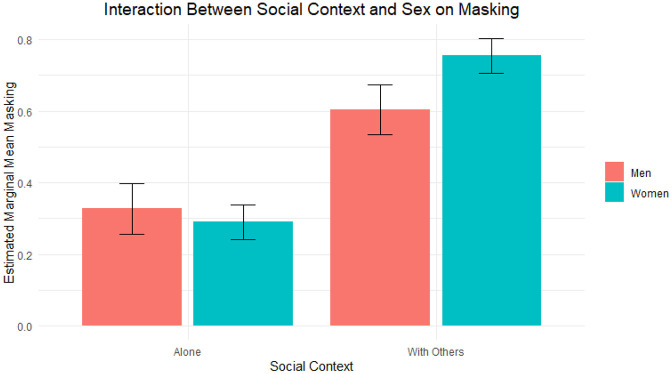
Interaction effect between social context and sex on masking.

Comparable with the prior LMM, there was a main effect of social context on masking (*b* = 0.28, *SE* = 0.03, *t*(4702) = 8.97, *p* < 0.001), with higher levels of masking observed in the “With Others” condition compared with the “Alone” condition. The main effect of sex was not significant (*b* = –0.04, *SE* = 0.086, *t*(79) = –0.44, *p* = 0.663), suggesting no overall differences in masking between males and females. However, sex significantly moderated the association between social context and masking (*b* = 0.19, *SE* = 0.04, *t*(4702) = 5.11, *p* < 0.001, see also Figure 1). Both males and females masked more in the “With Others” condition compared with the “Alone” condition (males: *b* = 0.27, *SE* = 0.029, *p* < 0.001; females: *b* = 0.46, *SE* = 0.02, *p* < 0.001), but this social context effect was stronger for females. Similar results were found when self-identified gender was added as a moderator instead of biological sex (see Table 2).

**Table 2.** Effects of social context and self-identified gender on masking.

Predictor	*b*	*SE*	*t*(*df*)	*p*
Intercept	0.33	0.08	4.22 (4701)	<0.001
Co-occurring conditions	0.01	0.08	0.14 (78)	0.888
Social context	0.26	0.03	8.69 (4701)	<0.001
Gender	–0.07	0.09	0.85 (78)	0.398
Social Context × Gender	0.21	0.04	5.59 (4701)	<0.001

For social context, the reference category is “alone.” Gender is based on self-identification, with “men” as the reference category. Data of those not identifying as men or women were included in the statistical model, but the effect of non-binary/other gender is not presented in the table due to the small sample size.

#### Second hypothesis: sex/gender moderation

Because of the small number of participants who reported on masking and perceived stress in the company of autistic others (*n* = 35), statistical power was deemed too low to assess moderation by sex or gender.

#### Third hypothesis: sex/gender moderation

An nbGLMM with sex as a moderator in the relation between masking and stress revealed no moderation effect (masking within: *b* = –0.04, *SE* = 0.05, *z* = –0.90, *p* = 0.370; masking between: *b* = –0.48, *SE* = 0.46, *z* = –1.05, *p* = 0.293), indicating a similar association between masking and stress for both sexes. Furthermore, females reported higher perceived stress scores (*M* = 4.48, *SD* = 4.21) compared with males (*M* = 3.61, *SD* = 3.90, *b* = 0.69, *SE* = 0.29, *z* = 2.37, *p* = 0.018). Similar results were found when gender was added as a moderator (see Table 3).

**Table 3.** Outcomes of nbGLMM for the effects of masking on stress with self-identified gender as a moderator.

Predictor	Estimate	*SE*	*z*	*p*
Intercept	0.96	0.15	6.41	<0.001
Co-occurring conditions	–0.09	0.16	–0.59	0.558
Masking (within)	0.44	0.04	11.38	<0.001
Masking (between)	1.60	0.37	4.36	<0.001
Gender	0.46	0.16	2.83	0.004
Masking (within) × Gender	–0.04	0.05	–0.80	0.424
Masking (between) × Gender	–0.54	0.50	–1.08	0.280

Gender is based on self-identification, with “men” as the reference category. Data of those not identifying as men or women were included in the statistical model, but the effect of non-binary/other gender is not presented in the table due to the small sample size. nbGLMM = negative binomial generalized linear mixed model.

#### Effect of masking on stress at the next timepoint (4 h later): sex/gender moderation

To investigate the effects of masking on perceived stress 4 h later, we performed an nbGLMM with participants as random intercept and sex/gender as a moderator in the relation between masking and stress 4 h later. Controlling for current masking, we found no within-subject effect of prior masking on perceived stress (*b* = 0.10, *SE* = 0.09, *z* = 1.14, *p* = 0.253), indicating that day-to-day variation in masking within participants (within-subjects effect) was not related to stress 4 h later. Between-subject differences in average masking levels were significantly associated with stress 4 h later (*b* = 1.14, *SE* = 0.41, *z* = 2.81, *p* = 0.005). This means that average masking per day was related to stress 4 h later. There was no significant moderation effect of sex on the association between masking and stress 4 h later (within: *b* = –0.07, *SE* = 0.10, *z* = –0.69, *p* = 0.50; between: *b* = –0.42, *SE* = 0.50, *z* = –0.83, *p* = 0.405). Consistent with sex differences in current stress, females consistently reported higher stress levels 4 h later (*b* = 0.43, *SE* = 0.18, *z* = 2.36, *p* = 0.018). Results of the nbGLMM were similar when self-identified gender was added as a moderator instead of biological sex (see Table 4).

**Table 4.** Outcomes of nbGLMM for the effects of masking on stress levels 4 h later with self-identified gender as a moderator.

Predictor	Estimate	*SE*	*z*	*p*
Intercept	0.68	0.17	3.92	<0.001
Co-occurring conditions	0.01	0.18	0.08	0.939
Masking 4 h later	0.41	0.04	10.93	<0.001
Masking (within)	0.03	0.08	0.32	0.751
Masking (between)	1.04	0.41	2.58	<0.001
Gender	0.46	0.18	2.52	0.011
Masking (within) × Gender	0.03	0.09	0.28	0.782
Masking (between) × Gender	–0.21	0.54	–0.39	0.694

Gender is based on self-identification, with “men” as the reference category. Data of those not identifying as men or women were included in the statistical model, but the effect of non-binary/other gender is not presented in the table due to the small sample size. nbGLMM = negative binomial generalized linear mixed model.

#### Masking and concurrent stress: moderation by autism traits

**Figure 2.**
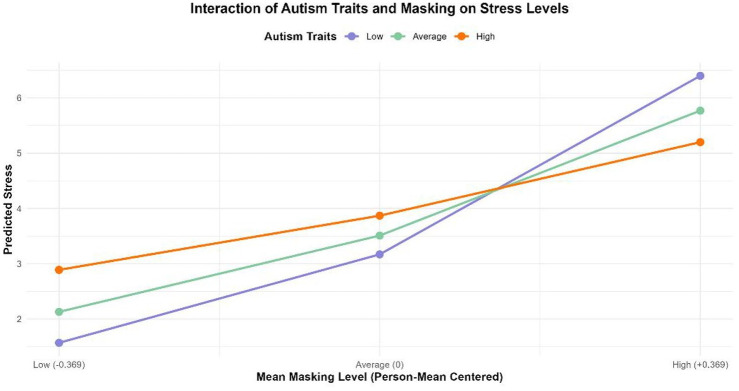
Interaction of autism traits and masking on stress levels.

In an additional exploratory analysis, we examined whether degree of autism traits, as measured with the AQ-28, moderated the relationship between masking and stress at the same time point. We found a significant interaction between person-mean masking and autism traits (*b* = –0.055, *SE* = 0.020, *p* = 0.006), indicating that the association between average masking levels and stress depended on an individual’s level of autism traits. No significant interaction was observed between moment-to-moment (within-person) masking and autism traits (*p* = 0.733). Model-based estimates showed that predicted stress increased with higher person-mean masking across all participants. However, this increase was most pronounced among individuals with fewer autism traits. For example, low AQ scores (–1 *SD*) predicted stress rose from 1.57 to 6.40 across masking levels, whereas at high AQ scores (+1 *SD*), predicted stress rose more modestly from 2.89 to 5.20 (see Figure 2). These predictions control for co-occurring conditions.

## Discussion

In this EMA study, we examined real-time associations between social context, masking and perceived stress in autistic adults in their everyday life. In line with our predictions, (1) autistic individuals reported that they engaged in less masking when they were alone compared with when others were present, (2) autistic individuals masked more when they were in the presence of non-autistic others as opposed to autistic others, and (3) when autistic individuals masked more in the past 4 h, higher levels of perceived stress were noted during that same time period. Linear mixed models (LMM) confirmed these findings. The positive association between masking and (both concurrent and subsequent) perceived stress was comparable across sexes/genders. Sex/gender did moderate the association between masking and social context (being alone vs. with others), with autistic women masking more than autistic men when with others. Finally, on an individual level, degree of masking at one timepoint was not associated with degree of perceived stress 4 h later.

The finding that autistic adults could be more themselves when alone compared with when they were in the company of others aligns with an abundance of research showing that both autistic and non-autistic people feel the need to act in a way that may not completely reflect their true selves when in social situations (Ai et al., 2022; Cook et al., 2024). The effect of social context on masking was even more pronounced among autistic women, corresponding with earlier reports of women masking slightly more than men (Cook et al., 2021). Interestingly, autistic adults masked less in the company of autistic others compared with non-autistic others or in groups of people from mixed neurotypes. This finding aligns well with empirical findings of improved social self-efficacy (Camus et al., 2024) and more self-disclosure (Morrison et al., 2020) during interactions among autistic individuals compared with interactions with people of mixed neurotypes. Autistic individuals may mask less in the company of autistic others, because of a shared understanding (Milton, 2012), sense of belonging, ease of communication, and acceptance of each other (Watts et al., 2024). Yet, in the company of non-autistic others, fear of rejection and stigmatization may fuel the need to mask (Ai et al., 2022; Bradley et al., 2021; Pearson & Rose, 2021; Perry et al., 2022).

In line with results from retrospective survey studies (Cook et al., 2021), more masking was associated with a higher level of perceived stress during that same time period, explaining 32% of variance. However, prior masking did not predict an individual’s perceived stress 4 h later, after controlling for current masking level. The positive association between masking and level of concurrent stress was similar across sexes/genders. Thus, even though autistic women in our sample reported masking more than men (when they were in the presence of others), their stress level was affected in the same way by masking as it was in men. The potential short-term mental health repercussions of masking may therefore be largely similar for both sexes/genders, and may depend more strongly on other factors such as degree of autism traits (van der Putten, Mol, Radhoe, et al., 2024). In our study, the association between masking and concurrent stress was indeed moderated by the degree of self-reported autism traits. Counter to findings in a general population sample (Ai et al., 2024), the stress difference between high and low maskers was most prominent among those who ascribed relatively few autism traits to themselves. Plausibly, those with few autism traits can mask more convincingly and therefore the alignment of the environment with their social, cognitive, and sensory needs may vary considerably depending on their masking level (e.g. the high masking group with few autism traits may be overburdened because their autistic needs are not recognized). Yet, people with many autistic traits may be less likely to completely mask their autistic traits and responses from the environment may therefore depend less on whether they generally are a high or low masker.

Together, these findings add to an accumulating body of evidence for the negative impact that masking potentially has on the mental health of (a subgroup of) autistic adults. How this mechanism works exactly is, as yet, unknown. We mainly focused on the impact of masking on psychological stress in this study, but it is likely that the link between masking and stress is bidirectional. Masking could lead to immediate stress, such as worrying about making a social faux pas. In qualitative reports, autistic individuals have described masking as very stressful, cognitively draining and resulting in a reduced sense of self (Bradley et al., 2021; Cook et al., 2024; Field et al., 2024), potentially contributing to mental fatigue and mental health problems. However, prior stressful interactions may also reinforce masking. For instance, experiences of being stigmatized as part of a neurodivergent minority in a predominantly non-autistic society may strengthen the urge to mask (Botha & Frost, 2020), thus perpetuating the link between masking, stress and poor mental health.

Our study findings underscore the value of specific places or events where autistic, or possibly broader neurodivergent, people can come together and freely express their authentic selves. Indeed, a systematic review of 52 qualitative studies indicated that for a majority of autistic people, contact with autistic others was beneficial to their emotional well-being (Watts et al., 2024). However, Watts et al. (2024) also mention that contact with other autistic people should not be viewed as a panacea because of the heterogeneity of autism. Also, positive connections with others may be driven more by mutual acceptance and understanding rather than a shared neurotype. In the reality of a neurodiverse society with a non-autistic majority, we believe this mutual acceptance of different needs and perspectives may be a particularly powerful route to reduce autistic masking and improve the mental well-being of autistic individuals.

The conclusions of this study should be considered in the context of its strengths and limitations. Prior research on autistic masking and mental health mainly relied on retrospective, cross-sectional reports, weakening any claims about causality. A unique strength of this study, therefore, is the use of EMA data, making it possible to examine real-time and temporal associations between social context, masking, and stress in everyday life. Other study strengths are the preregistration of our main hypotheses and analyses, as well as the co-creation of the stress monitoring application (SAM) together with autistic individuals. A study limitation is the operationalization of masking. Autistic masking is a complex construct consisting of multiple components such as compensation strategies and assimilation (Hull et al., 2019). However, given the time constraints of repeated assessments in EMA research, EMA surveys should preferably be short and easy to fill in. We, therefore, assessed masking with a single item concerning the degree to which participants felt they could be themselves. Many individuals with autism report the experience of not being their authentic self or being “fake” when they mask (Cook et al., 2021; Hull et al., 2017; Ridgway et al., 2024). Because our masking item (“being myself”) may cover a broader concept including other forms of masking one’s authentic self, such as hiding particular personality traits, sexuality, or gender identity, we cannot conclude with certainty that autistic masking in particular is associated with perceived stress. Yet, our finding of increased masking in the presence of non-autistic others does suggest that autistic masking is likely to be involved. Another limitation is the selectivity of our sample, including many late-diagnosed Dutch women with a higher education degree. The use of a digital stress monitoring application with verbal prompts and responses likely contributed to a sample of autistic individuals with average to high intellectual abilities, access to digital tools, and, possibly, a high need for stress relief as they all joined an RCT examining the effectiveness of an app on stress reduction (Spaargaren et al., 2025). Furthermore, the role of neurotype compatibility was a factor of interest in this masking study, but it is likely that many other contextual and intrapersonal factors contribute to autistic masking and its short- and long-term effects. Future (EMA) studies should expand on potential factors that influence masking and stress, such as the level of familiarity, stigmatization or acceptance by social partners, and the impact of different social norms for sexes/genders and cultures. EMA research offers a unique way to study the temporal dynamics of variables in a naturalistic setting, but the repetitive assessments may also increase awareness about the variables being assessed. This increased awareness may be a factor to consider and examine in future studies. Finally, potential positive consequences of masking (e.g. maintenance of social relations) were not addressed in this study, even though these may also be present.

This is the first EMA study showing positive real-time associations between (1) the presence of non-autistic others and degree of masking, and (2) masking and perceived stress in autistic adults. These findings provide ecological validity to reports of reduced masking among individuals with autism, and to the potential stressful impact of masking in everyday life. Less masking among autistic individuals compared with masking in the presence of non-autistics may stem from (expected) higher acceptance of autistic traits by the social environment. Consistent with a social model of autism, increased societal understanding and acceptance of neurodiversity and neurodivergence may be a promising route to reduce autistic masking and boost the mental health of autistic adults.

## Notes

1. We use the terms “autistic individuals” and “individuals with autism” interchangeably, so that it reflects the mixed preferences of adults with autism in the Netherlands (see Buijsman et al., 2022).
